# On Etherization

**Published:** 1847-06

**Authors:** 


*On Etherization.*—In our historical notices of the effects resulting from the use of ether, we have endeavored merely to record the facts as they arise. It would seem, however, that the article in our last number has led to some misapprehension. In alluding to the alleged fatal effects of this substance, we thought we had been sufficiently cautious. It was asked, " is this result, the effect of ether? an answer in the affirmative cannot be decidedly given, but all such cases require to be put on record." We continue to be of the same opinion, and shall put on record all the fatal cases that occur *after* the employment of ether, being satisfied that it is of the utmost consequence to ascertain whether it be innocuous or occasionally dangerous, and in the latter case, what are the contraindications to its employment. A correct judgment can only be formed by further experience and multiplied observations. We deprecate alike the excessive enthusiasm which insists that under no possible circumstances, ether can be, or ever has been prejudicial, and the unreasonable timidity which prevents the employment of a useful agent, because, in a few cases, injurious effects have been apparently occasioned by it.

During the past month Etherization has been extensively practised, but few novelties have been published with respect to it. Its advantages and applications are still debated at the meetings of the Academy of Natural Sciences and the Academy of Medicine in Paris. We observe, however, that the cases noticed in our last number have produced an effect on the warmest advocates of inhalation. Even MM. Velpeau and Roux, though still maintaining its great advantages, now speak of the necessity of caution in its use. This is as it should be.

The third case to which we alluded, as likely to be fatal *after* the use of ether, in the Royal Infirmary has since expired. It was a case of tibio-tarsal amputation, under the care of Mr. Syme. A girl, aged 14, of good general health, was affected with caries of the tarsal bones, and fistulous openings leading from them. The amputation was performed in the usual manner on the 24th of February, without the slightest pain, the ether having produced its full effect. She died April 5. On dissection the blood was found unusually fluid, and secondary purulent deposits existed in the lungs, left kidney, right knee, and left hip joint. Such are the *facts.* As to whether death in this, or the other two Infirmary cases, resulted from the etherization, that is a matter of *opinion.* Some say no, others say yes. It is the first fatal case of tarsal amputation which has occurred in Mr. Syme's practice, and it is only right to state that in his opinion it is attributable to the ether. The observation of other cases will sooner or later decide the point.

*Applications.*—In the case of a young man, aged 23 years, subject for some years to epileptic attacks, which returned every 15 days, M. de Rabodanges caused ether to be inhaled, the evening before the day on which the attack was expected, with the result of preventing it. (*L'Union Médicale*, No. 42.) M. Marc Dupuy has injected large doses of ether into the rectum of two dogs, and found that in this way it will cause perfect loss of sensation. Slight inflammation of the mucous membrane was produced in one case. (*Ibid*, No. 24.) M. Stolz, of Strasburgh, has published a case of turning, in which he met with considerable resistance, in endeavoring to pass his hand into the uterus, notwithstanding the complete insensibility of the patient, by means of ether. He concludes from it that ether in no way facilitates the turning or extraction of the fœtus.—*Monthly Jour. of Med. Sci., from Gaz. Med. de Stras.*

